# Rehabilitation interventions in bronchiectasis: expanding evidence, personalization, and clinical practice

**DOI:** 10.3389/fresc.2026.1785522

**Published:** 2026-03-25

**Authors:** Antonio M. Esquinas, Fernando Silva Guimaraes

**Affiliations:** 1 Hospital General Universitario Jose M Morales Meseguer, Murcia, Spain; 2 NIV-ICU Group, Biomedical Research Institute Pascual Parrilla, Murcia, Spain; 3 Rehabilitation Sciences Graduate Program and Faculty of Physical Therapy, Federal University of Rio de Janeiro, Rio de Janeiro, Brazil

**Keywords:** airway clearance techniques, bronchiectasis, exercise, non pharmacologic treatment, pulmonary rehabilitation

## Abstract

Bronchiectasis is a chronic and heterogeneous respiratory disease characterized by irreversible airway dilation, recurrent infection, persistent inflammation, and progressive functional impairment. Pulmonary rehabilitation is consistently recommended in international guidelines as a core non-pharmacological intervention; however, its implementation in clinical practice remains inconsistent and access to comprehensive rehabilitation programmes remains limited despite strong guideline endorsement. Recent advances in disease conceptualization, particularly the treatable traits framework, together with growing evidence supporting exercise training, physical activity promotion, and digitally enabled care models, have expanded the scope and relevance of rehabilitation in bronchiectasis. This Mini Review synthesizes contemporary evidence on rehabilitation interventions, integrating established practices with emerging strategies, and critically discusses current controversies, research gaps, and future directions. While airway clearance and exercise training remain foundational, current European Respiratory Society guidance supports their use based on defined patient profiles and evidence strength, and increasing evidence supports individualized, multimodal rehabilitation approaches that incorporate physical activity promotion, adjunct interventions, home-based and tele-rehabilitation models, and patient-managed strategies. Persistent uncertainties regarding optimal prescription, implementation, and long-term outcomes underscore the need for patient-centered, phenotype-informed rehabilitation strategies. Broadening rehabilitation beyond traditional paradigms may enhance clinical relevance, improve functional outcomes, and better align care with the complex and evolving nature of bronchiectasis.

## Introduction

Bronchiectasis is a chronic respiratory condition associated with recurrent exacerbations, chronic cough, sputum production, dyspnea, fatigue, and impaired health-related quality of life. Once considered a neglected disease, it has become increasingly prevalent globally due to improved access to high-resolution computed tomography, aging populations, and greater clinical awareness (1, 2). Contemporary perspectives recognize bronchiectasis as a heterogeneous condition encompassing diverse etiologies, phenotypes, and clinical trajectories rather than a single disease entity (3).

Updated international guidance emphasizes comprehensive and multidisciplinary management. The most recent European Respiratory Society (ERS) clinical practice guideline reinforces the central role of non-pharmacological strategies, particularly airway clearance techniques and pulmonary rehabilitation, and provides strong recommendations supported by moderate-to-high-quality evidence (4). Airway clearance techniques are recommended for patients with chronic sputum production, recurrent exacerbations, or radiological evidence of mucus plugging, whereas pulmonary rehabilitation is indicated for individuals with exercise intolerance, reduced physical activity levels, dyspnea, or impaired health-related quality of life, irrespective of disease severity (4). Despite these recommendations, referral to pulmonary rehabilitation programmes for people with bronchiectasis remains disproportionately low. Airway clearance techniques tend to receive greater clinical attention because they directly target one of the most burdensome and immediately perceptible symptoms of the disease, namely, chronic sputum production. In contrast, many pulmonary rehabilitation programmes have historically been structured around conditions such as COPD, which may limit their alignment with the specific clinical needs of people with bronchiectasis.

**Figure 1:**
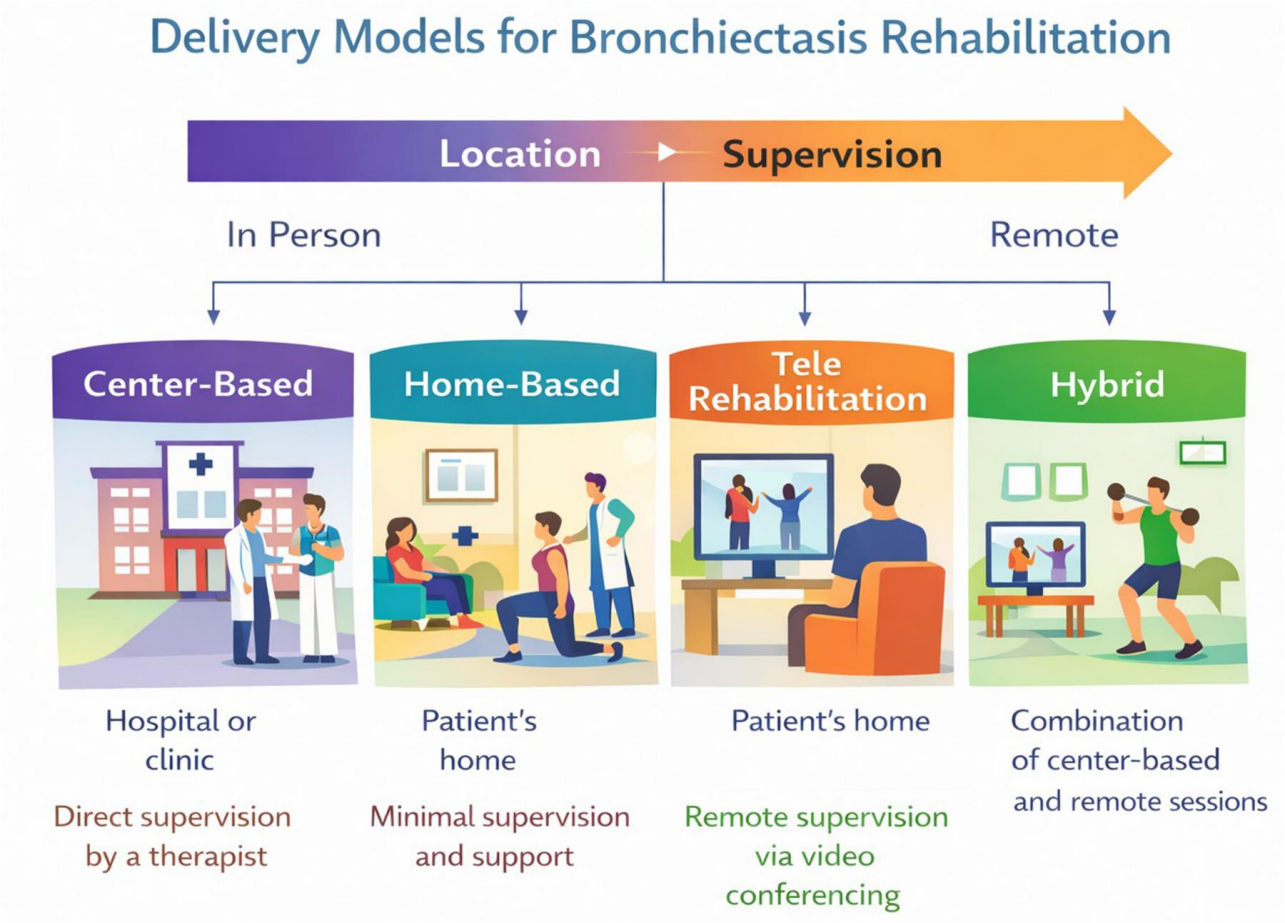
Organization of rehabilitation models for bronchiectasis. The diagram highlights the spectrum of care across different settings (clinical vs. home-based), distinguishing between direct professional supervision, minimal support, remote monitoring via videoconferencing (telerehabilitation), and mixed approaches (hybrid models).

The emergence of the treatable traits paradigm has challenged protocol-driven approaches by advocating individualized assessment and targeted interventions across pulmonary, extrapulmonary, and behavioral domains from a predominantly non-pharmacological perspective (5, 6). Within this framework, rehabilitation extends beyond conventional chest physiotherapy to encompass structured exercise training, promotion of physical activity, nutritional and frailty management, digital health solutions, and patient-managed strategies. The spectrum of care for bronchiectasis rehabilitation spans from in-person clinical sessions to patient-centered home-based strategies (Figure 1).

## Conventional rehabilitation interventions: current foundations

### Airway clearance techniques

Airway clearance techniques remain a cornerstone of bronchiectasis management, particularly for individuals with chronic sputum production or radiological evidence of mucus plugging, which has been explicitly recognized in the updated ERS guideline as an indication for airway clearance interventions (4, 7). The European Respiratory Society statement on airway clearance techniques emphasizes their role in facilitating mucus transport, reducing symptom burden, and supporting self-management (7).

Real-world data from the EMBARC registry and the European Lung Foundation (ELF) survey demonstrate substantial variability in access to airway clearance interventions and pulmonary rehabilitation, with many patients reporting limited availability of specialist physiotherapy despite strong guideline recommendations (8, 9). These findings highlight persistent gaps between evidence and implementation, particularly for non-pharmacological care.

Recent conceptual advances emphasize that airway clearance and exercise should be viewed as complementary rather than competing interventions (10). Nonetheless, controversies persist regarding routine vs. selective prescription, optimal frequency, and long-term adherence, particularly in patients with mild disease or limited sputum burden. Mucoactive agents, particularly hypertonic saline solutions, are frequently used in clinical practice as adjuncts to airway clearance techniques; however, recent negative findings from the CLEAR trial have raised uncertainty about their long-term clinical impact, underscoring the need for individualized decision-making.

High-flow nasal therapy may be considered as an adjunct to airway clearance techniques in selected patients with advanced disease, mucus retention, or poor tolerance to conventional techniques, although its role within rehabilitation frameworks remains to be clearly defined (11).

### Exercise training and pulmonary rehabilitation

Exercise intolerance is a prominent feature of bronchiectasis and is driven by ventilatory limitation, peripheral muscle dysfunction, systemic inflammation, and comorbidities. Structured exercise training delivered within pulmonary rehabilitation programmes consistently improves exercise capacity, dyspnea, and health-related quality of life, with effects comparable to those observed in COPD and strong support in international guidelines (12–14).

Beyond symptomatic benefit, emerging evidence supports the prognostic relevance of physical activity. Higher habitual physical activity levels are associated with reduced all-cause mortality in individuals with bronchiectasis (14). In addition, sedentary behaviour has been independently associated with an increased risk of hospitalisation, and exacerbations are known to negatively impact physical activity behaviour, further reinforcing the importance of physical activity promotion as a core rehabilitation target (15, 16).

**Figure 2:**
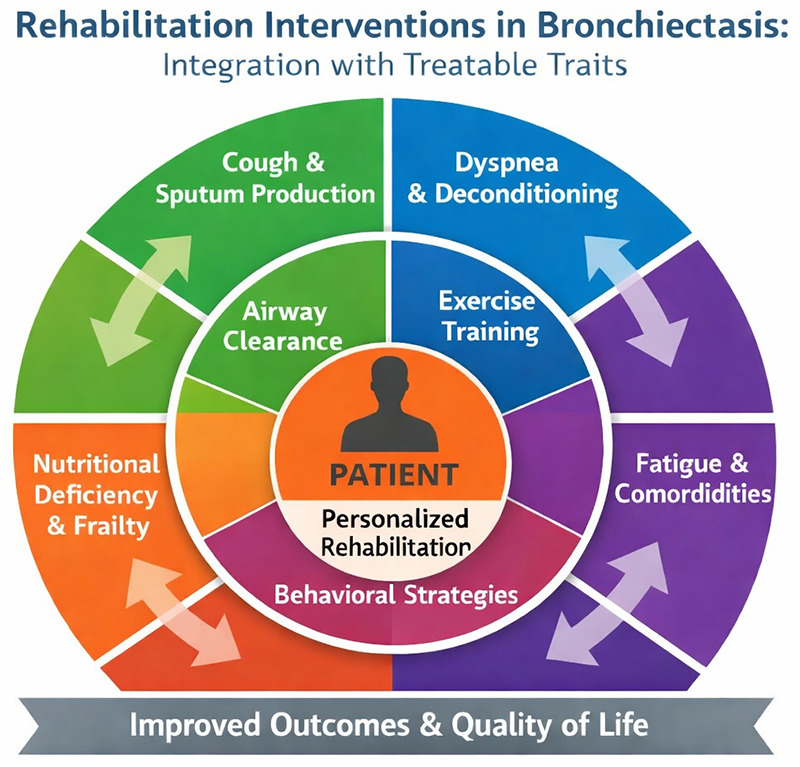
Integration of rehabilitation interventions with treatable traits in bronchiectasis. The diagram centers the patient within a personalized rehabilitation model, linking specific interventions (airway clearance, exercise training, and behavioral strategies) to corresponding clinical traits (cough, dyspnea, fatigue, and frailty) to optimize clinical outcomes and quality of life.

Despite robust evidence, participation in center-based pulmonary rehabilitation remains suboptimal. Data from the ELF survey confirm that access to pulmonary rehabilitation programmes for people with bronchiectasis remains extremely low, despite strong guideline endorsement, and real-world implementation continues to lag behind evidence (4, 9, 13). Rehabilitation interventions should be tailored to the patient's specific clinical traits, integrating airway clearance and exercise training to optimize outcomes (Figure 2).

## Emerging and adjunct rehabilitation strategies

### Home-based and digital rehabilitation models

Home-based pulmonary rehabilitation has emerged as a viable alternative to conventional center-based programs. Randomized trials and feasibility studies indicate that home-based models can achieve clinically meaningful improvements in functional outcomes and health-related quality of life, with acceptable safety profiles (17, 18).

Digital health innovations have further expanded access to rehabilitation. Smartphone app-based programs, video consultations, and hybrid tele-rehabilitation models have demonstrated feasibility and short-term effectiveness in chronic respiratory diseases, including bronchiectasis (19–22). Surveys of patients and physiotherapists suggest increasing acceptance of digital tools, although challenges remain related to digital literacy, long-term engagement, and integration into routine clinical services (9, 22).

### Inspiratory muscle training and adjunct therapies

Inspiratory muscle training has been explored as an adjunct intervention, particularly in patients with inspiratory muscle weakness or disproportionate dyspnoea. However, the current evidence supporting inspiratory muscle training in bronchiectasis remains limited, and this intervention is not considered an essential component of pulmonary rehabilitation programmes, consistent with recommendations in other chronic respiratory diseases (23). Inspiratory muscle training may be considered in carefully selected patients who are unable to participate in structured exercise or pulmonary rehabilitation programmes due to comorbidities, limited digital literacy, or restricted access to hospital-based services.

### Addressing frailty, multimorbidity, and comorbidities

Bronchiectasis predominantly affects older adults, many of whom present with frailty, sarcopenia, and multiple comorbidities. Cardiovascular comorbidities are highly prevalent in people with bronchiectasis and can significantly affect exercise capacity, recovery after exertion, and overall functional performance, representing an important but under-recognized challenge for rehabilitation planning (24).

## Controversies and divergent perspectives

Several unresolved issues continue to shape rehabilitation practice in bronchiectasis. One major controversy concerns whether airway clearance techniques should be universally prescribed or selectively targeted based on clinical phenotype and patient-reported benefit. While international guidelines strongly support airway clearance in patients with chronic sputum production and radiological evidence of mucus plugging, uncertainty remains regarding their routine use in individuals with mild disease or limited sputum burden, highlighting the need for individualized assessment and periodic reassessment (4, 7).

Similarly, there is no consensus on the optimal intensity, duration, or modality of exercise training, nor on the most appropriate timing of pulmonary rehabilitation across the disease course. Although pulmonary rehabilitation is strongly endorsed by European and international guidelines, real-world access remains limited, and programmes are often not tailored to the specific needs of people with bronchiectasis, contributing to underutilization despite robust evidence of benefit (4, 9, 13).

The treatable traits framework has further intensified debate around standardized vs. personalized rehabilitation approaches. While standardized programmes facilitate scalability and implementation, increasing evidence supports individualized, trait-driven rehabilitation strategies that integrate airway clearance, exercise training, physical activity promotion, and management of extrapulmonary traits such as frailty and comorbidities (5, 6, 24). In this context, patient-managed interventions and shared decision-making challenge traditional, protocol-driven models by emphasizing autonomy, adaptability, and long-term engagement (20).

## Current research gaps

Despite expanding evidence, significant research gaps remain. Many studies prioritize short-term physiological outcomes, with limited emphasis on participation, autonomy, and patient-defined success. The recent development of a core outcome set for physiotherapy trials in bronchiectasis represents an important step toward improving the relevance and comparability of future research (25).

Additional key research gaps include: (i) the role and optimal prescription of airway clearance techniques during exacerbations; (ii) the effectiveness and timing of pulmonary rehabilitation during or immediately after exacerbations; and (iii) strategies to enhance the synergistic effects of exercise training and airway clearance techniques while minimizing overall treatment burden.

## Discussion

Rehabilitation interventions are central to contemporary bronchiectasis management, offering meaningful benefits across functional, symptomatic, and quality-of-life domains. However, current practice continues to underutilize the full potential of rehabilitation, with persistent gaps between strong guideline recommendations and real-world implementation, particularly regarding access to pulmonary rehabilitation programmes and comprehensive physiotherapy services (4, 9).

Recent advances, including updated European Respiratory Society guidance, the treatable traits paradigm, and digital health innovations, support a broader and more individualized approach to rehabilitation. Integrating airway clearance techniques with exercise training, physical activity promotion, and selected adjunct interventions may enhance synergistic effects while minimizing overall treatment burden, an area that remains insufficiently explored in current research.

The high prevalence of multimorbidity, particularly cardiovascular comorbidities, further underscores the need for personalized rehabilitation strategies. Exercise prescription and monitoring may require adaptation in patients with complex multimorbidity profiles to ensure safety, optimize adherence, and maximize functional gains, representing an important clinical and research priority (24).

Future research should prioritize patient-centered outcomes, implementation science, and long-term effectiveness across diverse healthcare settings. Clarifying the roles of airway clearance and pulmonary rehabilitation during and after exacerbations, and identifying strategies to optimize the combined delivery of exercise and airway clearance, may help reduce treatment burden and improve the sustainability of care. Broadening rehabilitation beyond traditional paradigms has the potential to enhance clinical relevance, improve outcomes, and better align care with the heterogeneous and chronic nature of bronchiectasis.
